# Temporal Variation in Target Site Mutations Is Associated with Diamide Cross-Resistance in Diamondback Moth Populations (Lepidoptera: Plutellidae) from Florida and Georgia, USA

**DOI:** 10.3390/insects16111179

**Published:** 2025-11-19

**Authors:** Thomas P. Dunn, Md. Abdullah Al Baki, Paulo S. G. Cremonez, David G. Riley, Alton N. Sparks, Hugh Smith, Donald E. Champagne

**Affiliations:** 1 Department of Entomology, University of Georgia, 418 Building C, 136 Cedar Street, Athens, GA 30602-2603, USA; thomas.dunn@uga.edu (T.P.D.); mdabdullah.albaki@uga.edu (M.A.A.B.); 2 Department of Entomology and Plant Pathology, Auburn University, 301 Funchess Hall, Auburn, AL 36849-5413, USA; psg0021@auburn.edu; 3 Department of Entomology, University of Georgia Tifton Campus, Bldg 4603, 110 Research Way, Tifton, GA 31794, USA; dgr@uga.edu (D.G.R.); asparks@uga.edu (A.N.S.J.); 4 Department of Entomology and Nematology, Gulf Coast Research and Education Center, University of Florida, 14625 CR 672, Wimauma, FL 32611, USA; hughasmith@ufl.edu

**Keywords:** *Plutella xylostella*, diamondback moth, diamides, I4790K, G4946E

## Abstract

The Diamondback moth (DBM), *Plutella xylostella* (L.), the most significant worldwide pest of *Brassica* crops, is notorious for resistance to a diverse number of insecticides. Field populations bioassayed in Georgia and Florida, USA, in 2018 were resistant to chlorantraniliprole but susceptible to cyantraniliprole. Subsequently, populations assayed in 2021, 2022, and 2023 were cross-resistant to both diamides. We used NextSeq analysis of the ryanodine receptor *PxRyR*, the target of diamides, to quantify target site mutations associated with resistance. Three populations sampled in 2018 had a high prevalence (75.0–98.3% of total reads) of the G4946E mutation, associated with resistance to chlorantraniliprole, and additionally, in one population, a very low (2.7%) prevalence of another mutation, I4790K, was associated with diamide cross-resistance. Populations sampled in 2021 had a decreased prevalence of G4946E (0.7 and 8.4%) and increased prevalence of I4790K (9.3 and 18.0%). The G4946E allele was almost absent (0.2% to 3.9%) in populations sampled in 2022 and 2023, while I4790K was present at frequencies from 34.1% to 84.0%. These data suggest a remarkable shift in *PxRyR* target site mutations, replacing G4946E with I4790K, which occurred between 2018 and 2022, and were associated with the recent occurrence of cross-resistance in DBM populations in the Southeastern USA.

## Simple Summary

The Diamondback moth (DBM), *Plutella xylostella* (L.), the most significant worldwide pest of *Brassica* crops, is notorious for its resistance to a diversity of insecticides. In Georgia and Florida, USA, control failures indicate that resistance to diamide insecticides is an increasing problem. We bioassayed field populations in 2018, 2021, 2022, and 2023 with two diamides: chlorantraniliprole and cyantraniliprole. In 2018, DBMs were resistant to chlorantraniliprole but susceptible to cyantraniliprole. However, populations assayed in 2021, 2022, and 2023 were cross-resistant to both diamides. We used sequencing of the ryanodine receptor *PxRyR*, the target of diamides, to quantify target site mutations associated with resistance. In 2018, the G4946E mutation, associated with resistance to chlorantraniliprole, was abundant in all populations, and a second mutation, I4790K, associated with diamide cross-resistance, was present but rare in one population. In 2021, G4946E was significantly decreased and I4790K increased. G4946E was almost absent in populations sampled in 2022 and 2023, while I4790K became dominant. These data suggest a remarkable shift in target site mutations, replacing G4946E with I4790K, which occurred between 2018 and 2022, and is associated with the development of cross-resistance in DBM populations in the Southeastern USA.

## 1. Introduction

The diamondback moth (DBM), *Plutella xylostella*, is a major specialist pest of cruciferous crops [1,2], and is the primary pest of *Brassica* crops in the Southeastern U.S. On a global scale, DBMs have consistently been an economic hurdle in vegetable production, with annual losses exceeding billions of dollars [1,3]. While research efforts have most recently been focused on identifying alternative control methods, insecticides are an essential tool of integrated pest management (IPM) in the Southeastern U.S. and are currently the most common tactic for the control of DBM infestations. The ability to rapidly develop resistance to multiple insecticides has resulted in global notoriety, as DBMs are now considered to be the most insecticide-resistant arthropod pest to date [4].

Diamide insecticides (IRAC Class 28), which target the ryanodine receptor (RyR), have been favored for DBM control since their registration during the mid-2000s [5,6,7,8]. The RyR is a calcium channel within the sarcoplasmic reticulum membrane that regulates the flow of Ca^2+^ ions into the sarcoplasm [9,10,11,12]. Once bound, diamides induce a conformational change, leaving the RyR in the open conformation. This causes an efflux of Ca^2+^ ions from the sarcoplasmic reticulum, which leads to the disruption of calcium homeostasis, ultimately resulting in feeding cessation, uncontrolled muscular contractions, and death [9,10,11,12,13]. The earliest reports of diamide resistance in DBM populations occurred in Thailand during the late 2000s, when resistance to flubendiamide was documented [14]. Subsequently, resistance to another diamide active ingredient, chlorantraniliprole, was reported in DBM populations from China in the early 2010s [5]. Resistance to chlorantraniliprole and flubendiamide persisted throughout the 2010s, while cross-resistance between flubendiamide, chlorantraniliprole, cyantraniliprole, cyclaniliprole, and tetraniliprole has occurred more recently in DBM populations from Asia [15,16,17].

Further studies of diamide insecticide resistance in Asian DBM populations have identified potential molecular mechanisms of resistance, with target site mutations of the RyR emerging as the most consistently documented mechanism. Troczka et al. [18] identified the G4946E mutation in flubendiamide- and chlorantraniliprole-resistant populations from Thailand (ThaiR) and the Philippines (Sudlon). Three additional target site mutations, E1338D, Q4594L, and I4790M, were later identified in chlorantraniliprole-resistant DBM populations from China [19]. Additionally, an I4790K mutation was discovered in DBM populations from Japan [15] and later identified in Australia [20]. While all discovered mutations seem to be associated with chlorantraniliprole and flubendiamide resistance, only the I4790K mutation has been identified in populations exhibiting high levels of cross-resistance between all diamide active ingredients [15,16,17]. Furthermore, mutations occurring at the G4946 and I4790 sites are more strongly associated with high levels of diamide resistance in DBM populations than those occurring at the E1338 or Q4594 sites [15,16,17,18,21,22,23]. Mutations corresponding to G4946E and I4790K/I4790M have also been reported in diamide-resistant populations of other insect species, which further supports the proposed importance of these two RyR mutation sites [24,25,26,27,28,29,30,31,32,33,34].

During the late 2010s, chlorantraniliprole resistance was consistently documented in DBM populations from Georgia and Florida [6,7,35], while as recently as 2019, susceptibility or lower levels of resistance to cyantraniliprole and cyclaniliprole were found in these states [6,7,35]. Subsequently, repeated instances of cross-resistance between the maximum labeled doses of chlorantraniliprole, cyantraniliprole, and cyclaniliprole were observed in DBM populations from Georgia and Florida from 2021 to 2023 [8,36]. While the G4946E mutation has been documented in DBM populations from the Southeastern U.S. [21,35], this mutation is primarily associated with resistance to flubendiamide, chlorantraniliprole, and tetraniliprole [16,18,35,37]. The recent changes in insecticide resistance, as well as preliminary molecular analysis, indicated that I4790K may be present in DBM populations from Georgia [38], and warranted investigation into the current mechanisms of diamide insecticide resistance in the Southeastern U.S. The current study provides additional evidence of cross-resistance among diamide insecticides in DBM populations from the Southeastern U.S via dose–response and maximum dose assays. Likewise, this study also provides evidence that the I4790K mutation has become a major mechanism of diamide resistance in the Southeastern U.S., while the G4946E mutation has become infrequent in these populations.

## 2. Materials and Methods

### 2.1. Insects

**Figure 1:**
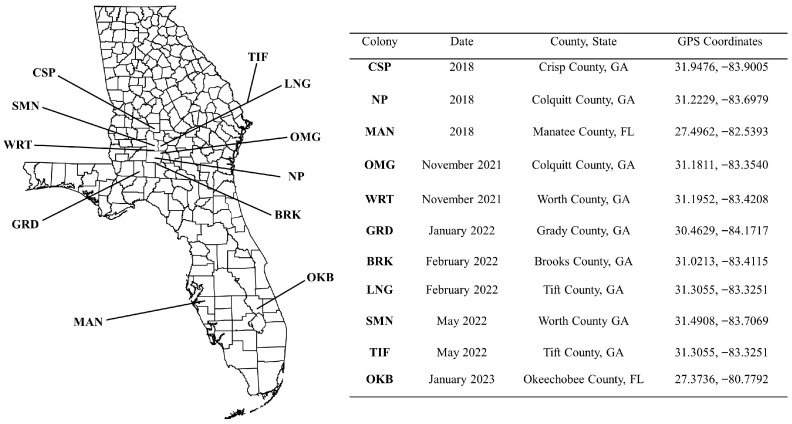
Collection sites, dates, and coordinates for all diamondback moth populations in this study.

A minimum of 200 *Plutella xylostella* larvae and pupae were collected from field populations in Colquitt (OMG colony), Worth (WRT colony and SMN colony), Grady (GRD colony), Brooks (BRK colony), and Tift (LNG colony and TIF colony) counties in Georgia from November 2021 to May 2022. Additionally, 100 larvae were collected from a DBM population from Okeechobee (OKB colony) County, Florida, in January of 2023. Collection sites are shown in Figure 1. A commercially available susceptible strain from Frontier Agricultural Sciences (Newark, DE, USA) (FRT colony) was used as a control and subject to the same toxicological and molecular analysis as our selected field populations. Each population was maintained as a laboratory colony, using previously described conditions [35]. First generation (F^1^), second to third instar larvae from each colony were used for bioassays and genetic analysis. The SMN and TIF colonies were collected in May of 2022 and maintained as laboratory colonies (as in [35]), and F^1^ larvae were used for dose–response assays to more accurately assess the observed resistance to diamide insecticides. These populations were not included in the sequencing analysis.

For populations from 2018, archived larval samples from previously established colonies were used for genetic analysis. These colonies originated from field populations in Colquitt (NP) and Crisp (CSP) counties in Georgia, as well as Manatee (MAN) county in Florida, and exhibited high levels of chlorantraniliprole resistance during the 2018 growing seasons. The dose–response data for these colonies were included in a previous study, alongside preliminary genetic analyses [35]. However, the corresponding maximum dose data was not reported for each population during that study and are reported here for the first time, whereas the dose–response data for those colonies serve only for comparison to the SMN and TIF populations. Additionally, the Illumina sequencing analysis for these colonies allows for more precise quantification of the target site mutation prevalence compared to our previous Sanger sequencing method and is reported here for the first time.

Each colony was collected from collard (*Brassica oleracea* var. *viridis*) fields, and laboratory colonies were established at the Coastal Plains Experiment Station in Tifton, Georgia. All collection dates, counties, states, and coordinates are detailed in Figure 1. Collard seedlings, grown in Percival growth chambers (Percival Scientific, Perry, IA, USA) at a temperature of 26.0 ± 1 °C, were used as a food source for the colonies. The day and night cycle for growing collard seedlings was 12:12. Adult DBM were given a 10% honey solution via soaked cotton balls placed in 37.0 mL SOLO cups. DBM colony cages were maintained with ambient lighting, and the room temperature was held constant at 21–24 °C.

### 2.2. Toxicological Bioassays

Leaf-dip bioassays were used to assess the levels of resistance to each of the diamide active ingredients at the maximum labeled dose. Chlorantraniliprole (Coragen ^®^, FMC Corporation, Philadelphia, PA, USA) and cyantraniliprole (Exirel ^®^, FMC Corporation, Philadelphia, PA, USA) were selected for bioassay of the FRT, OMG, WRT, GRD, LNG, BRK, and OKB colonies. In addition to maximum dose assays, dose–response assays were also conducted for chlorantraniliprole and cyantraniliprole for the SMN and TIF colonies. Additional F^1^ larvae from colonies subject to maximum dose bioassays were collected in microcentrifuge tubes containing 0.75 mL of RNAlater and stored at −40 °C until they were used for cDNA library preparation.

For the bioassay, untreated collard leaves grown to greater than seven weeks of age on bench tops in a greenhouse were used as the substrate. A 70 mm (2–3/4 inch) leaf disc was excised from the leaf and subsequently dipped in a 250 mL solution containing the appropriate insecticide for 10 s, as well as 0.25 mL (1000 ppm) of Kinetic adjuvant (Helena Agri-Enterprises, Collierville, TN, USA). For the maximum dose data, insecticide concentrations were equivalent to their respective maximum labeled rates as applied at 100 gallons per acre and are as follows: Coragen (7.5 oz/acre) and Exirel (13.5 oz/acre). Dose–response assays were completed for chlorantraniliprole and cyantraniliprole for the SMN and TIF colonies using the same protocol as the maximum dose assays. Concentrations of chlorantraniliprole were 0.78, 7.8, 78, 156, 312, and 468 milligrams of active ingredient per liter, as well as an untreated control. Assayed concentrations of cyantraniliprole included 10.5, 105, 210, 420, and 630 milligrams of active ingredient per liter, as well as an untreated control.

After treating the leaf disc with the appropriate concentration, leaves were air dried for 30 to 60 min and then were placed in the Petri dish onto circular Whatman filter papers (90 mm) (Cytiva, Marlborough, MA, USA), which had been dampened to prevent leaf disc desiccation. The lids of the Petri dishes had 38 mm (1–1/2 inch) holes cut for improved ventilation, as well as hot-glued nylon chiffon to prevent escape from the arena. Ten 3rd instar larvae were placed on the treated leaf disc, and the Petri dish was secured with rubber bands. This method was repeated in a minimum of triplicate for each treatment, and live, dead, moribund, and pupated larvae were recorded every 24 h until reaching the 72 h mark. A straight tip teasing probe (Bioquip^®^, Rancho Domingues, CA, USA) was used to differentiate responses of live (rapid reaction), moribund (lethargic reaction), and dead (no reaction) individuals.

### 2.3. mRNA Extraction and cDNA Synthesis

For molecular analysis, larval samples were collected at the time of each bioassay to accurately represent the frequency of each target site mutation in each population. Our samples included the previously mentioned OMG, WRT, GRD, BRK, LNG, and OKB populations collected from 2021 to 2023, the MAN, CSP, and NP populations collected from 2018 [35], and the highly susceptible FRT population. Third instar larvae from each population were excised at the midsection, and the anterior half of each individual was pooled into samples of 20 for mRNA extraction. Overall, samples consisted of three replicates per population with a total of 60 larvae (120 alleles) per population. For each replicate, mRNA extractions were completed using a Dynabeads mRNA Direct Kit (Invitrogen, Thermo Fisher Scientific, Waltham, MA, USA) following the manufacturer’s protocol. Subsequently, cDNA synthesis was completed using a VILO Superscript IV with EzDNAse kit (Invitrogen) following the manufacturer’s protocol. Each cDNA sample was stored at −80 °C in Athens, GA, USA, until used as a template in polymerase chain reaction (PCR).

### 2.4. cDNA Library Preparation and Illumina Sequencing

A BIO-RAD C1000 Touch (Life Science, Hercules, CA, USA) thermal cycler was used to prepare cDNA libraries for Illumina sequencing. PCR was completed using Phusion Green High-Fidelity Polymerase and 5X HF Reaction Buffer (Thermo Fisher Scientific) following the manufacturer’s protocol. Primer pairs, designed using Primer3Plus, generated two separate amplicons spanning the I4790 and G4946 mutation sites. Additionally, these primer pairs were designed with the overhang sequences specific to the Illumina sequencing platform and are detailed in Table S1. PCRs were completed independently with both primer pairs for all 30 samples, generating a total of 60 samples. Run specifications such as annealing temperatures, extension times, number of cycles, and amplicon size are included in Table S2. Subsequently, the size of the products for each sample was analyzed using a 1.5% agarose gel via gel electrophoresis. Only samples of the expected size were subjected to paired-end (PE) 300 sequencing using the Illumina NextSeq platform (Illumina, San Diego, CA, USA), utilizing the P1 flow cell. Sample indexing and Illumina Next Generation Sequencing (NGS) using the NextSeq platform was completed at the Georgia Genomics and Bioinformatics Center (GGBC) at the University of Georgia in Athens, Georgia. Raw sequencing data were then curated via Galaxy (usegalaxy.org). Reads were groomed via FASTQ Groomer, followed by mapping both forward and reverse reads to a consensus sequence (Accession: JF927788) via the BWA-MEM function in Galaxy. Integrative Genome Viewer (IGV) (Broad Institute, Cambridge, MA, USA) was used to view sequencing results in the generated BAM files and provide base pair frequencies for each mutant/susceptible allele.

### 2.5. Statistical Analysis

The dose–response data were analyzed in SAS Enterprise Guide 8.3 via PROC-PROBIT analysis to generate LC_50_ values of chlorantraniliprole and cyantraniliprole for the SMN and TIF colonies (Table 1). Maximum dose bioassay results for the FRT, MAN, NP, CSP, LNG, GRD, BRK, OMG WRT, and OKB colonies were analyzed in R Studio 4.5.1 (Posit, Boston, MA, USA) (Table 2). The Kruskal–Wallis One-Way ANOVA on Ranks was selected to analyze both datasets. Analysis was completed by comparing the treatment mortality of the susceptible FRT population to the treatment mortality of all other populations. This analysis was completed separately for each insecticide to identify statistical differences in percent mortality for each population. Following the ANOVA, Dunn’s post hoc test, alongside Benjamini–Hochberg correction, was completed to compare the percent mortality estimates. Significance was established if *p* < 0.05, while marginal differences were recorded at *p* < 0.1. An additional Kruskal–Wallis One-Way ANOVA on Ranks was completed to compare insecticide-induced mortality when populations were grouped according to collection dates. The groupings consisted of the FRT colony responses to both insecticides, the MAN, NP, and CSP colonies’ responses to both insecticides (2018), and the OMG, WRT, LNG, GRD, BRK, and OKB colonies’ responses to both insecticides (2021–2023). Once again, significance was established if *p* < 0.05, while marginal differences were recorded at *p* < 0.1.

**Figure 2:**
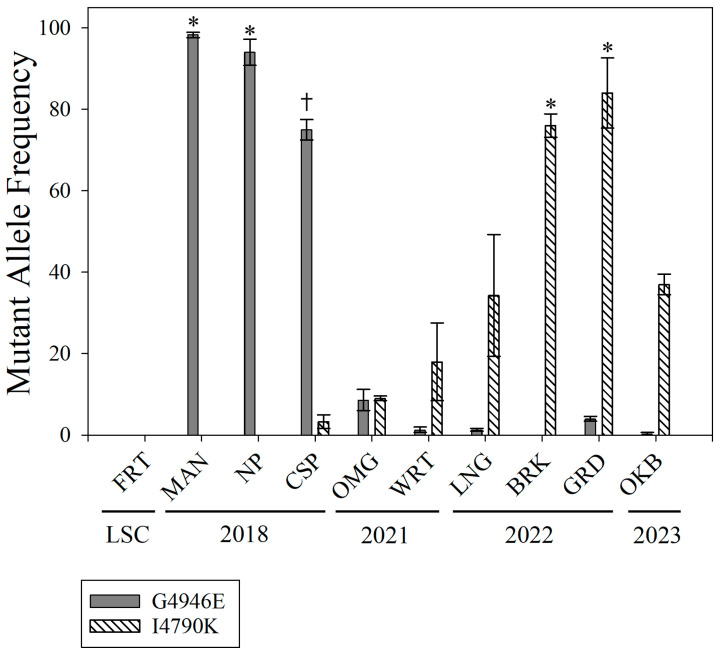
This figure depicts the I4790K and G4946E allele frequencies for all diamondback moth populations collected from 2018 to 2023. Statistical significance (*) is indicated at *p* < 0.05, while marginal differences (†) are indicated at *p* < 0.1.

For sequencing data, averaged allele frequencies from the Illumina NextSeq results were also analyzed via Kruskal–Wallis One-Way ANOVA on Ranks in R Studio. These analyses were completed separately by mutation to identify statistical differences in allele frequency between each population. As completed for the bioassay data, Dunn’s post hoc test was completed alongside Benjamini–Hochberg correction to compare the mutation percentage of both mutations in each population to the mutation percentage of the susceptible FRT population. Following the Kruskal–Wallis ANOVA, SigmaPlot SigmaStat (SPSS) Version 16.0 (Grafiti LLC, Palo Alto, CA, USA) was used to generate correlation coefficients (Spearman Rank Order Correlation) for estimates of each mutation (I4790K or G4946E) alongside the percent mortality recorded for the maximum labeled dose of either chlorantraniliprole or cyantraniliprole. Following statistical analysis, SPSS was used to generate a bar chart of the average allele estimates from the three replicates for each population (Figure 2).

## 3. Results

### 3.1. Dose–Response Assays

LC_50_ values were determined for cyantraniliprole and chlorantraniliprole for two field-derived colonies, SMN and TIF. The resistance ratios (RRs) were calculated relative to previously determined LC_50_ values for a field-derived colony (T-S) assayed in 2013 (reported in [6]), a susceptible laboratory colony (FRT), and three field-derived colonies collected in 2018 (CSP, NP, and MAN) (reported in [35]) (Table 1).

The SMN colony had a resistance ratio (RR) of 1582 for cyantraniliprole, relative to the susceptible FRT colony (Table 1). This was similar to an RR of 1416.9 when compared to an LC_50_ for the T-S colony. In comparison, RRs of 31.8, 23.6, and 14.7 were calculated relative to LC_50_ values for the 2018 CSP, NP, and MAN populations, respectively. The SMN population was also highly resistant to chlorantraniliprole relative to the susceptible FRT colony (RR = 2942.7), as well as the T-S population from 2013 (RR = 211.3). When compared to the 2018 colonies, the SMN population was clearly more resistant to chlorantraniliprole than the CSP population (RR = 27.0), but it did not differ from NP and MAN (RR = 0.7 and 1.0, respectively).

A similar pattern was found for the TIF population (Table 1), where almost identical RRs were found for cyantraniliprole in comparison to the FRT and T-S populations (RR = 382.4 and 342.2, respectively). However, only a modest increase in resistance was observed in comparison to the LC_50_ values for the 2018 CSP, NP, and MAN populations (RR = 7.7, 5.7, and 3.6, respectively). The TIF population was also highly resistant to chlorantraniliprole when compared to the FRT population (RR = 511.0), although TIF displayed intermediate levels of resistance in comparison to the T-S population (RR = 36.7). Comparisons to the 2018 colonies indicated higher levels of resistance to chlorantraniliprole relative to CSP (RR = 4.7), yet higher susceptibility relative to the NP and MAN populations (RR = 0.1 and 0.2, respectively).

### 3.2. Maximum Dose Bioassays

The results of the dose–response assays were recapitulated with the maximum dose bioassay results, as these indicated high levels of cross-resistance between chlorantraniliprole and cyantraniliprole occurred in populations collected after 2021 (Table 2). In contrast, the samples that had been assayed during our 2018 experiments displayed varying levels of resistance to chlorantraniliprole at the maximum dose, but retained susceptibility to cyantraniliprole (Table 2). When comparing the percent mortality of the FRT colony to each population, the results of the Kruskal–Wallis ANOVA by insecticide indicated significant differences occurred between percent mortality of the populations for chlorantraniliprole (H = 19.95, 9, *p* = 0.0182) and cyantraniliprole (H = 27.97, 9, *p* = 0.0009) (Table 2). Dunn’s post hoc test indicated that CSP and OKB were not significantly different from FRT regarding chlorantraniliprole resistance, while all other comparisons yielded marginally different results in comparison to FRT (Table 2). As for cyantraniliprole, the CSP, MAN, and NP colonies were not significantly different from FRT, while OKB was marginally different, and the OMG, WRT, LNG, GRD, and BRK colonies were significantly different (Table 2). For the Kruskal–Wallis One-Way ANOVA comparing the colonies based on time of collection, the ANOVA indicated there were significant differences among these data (H = 42.77, 5, <0.001) (Table 3). The 2018 colonies were still only marginally different from the FRT colony regarding chlorantraniliprole-induced mortality, but were not significantly different from FRT regarding cyantraniliprole mortality. For the 2021–2023 colonies, there was a statistically significant difference in mortality in comparison to FRT for both insecticides. Finally, when comparing the 2018 colonies to those collected from 2021 to 2023, chlorantraniliprole mortality was not significantly different, while cyantraniliprole mortality was significantly different (Table 3).

### 3.3. Mutation Frequency Analysis

Illumina Next Generation paired-end sequencing was used to quantify two target-site mutations, G4946E and I4790K, in the FRT susceptible colony, and the nine field populations: CSP, MAN, NP, OMG, WRT, LNG, GRD, BRK, and OKB. The NGS results generated a total of 132 million reads with 84% at or above Q30. Amplicon sequencing revealed varying frequencies of each mutant allele in the sampled populations (Figure 2). Averaged percentages for both mutations in each population are listed in Table S3. Counts for each of the four possible bases (ACGT) at the mutation site for each of the population samples are also reported in Table S4 for the *PxRyR* G4946E site and Table S5 for the I4790K site. Neither mutation was detected in the susceptible FRT laboratory colony samples. Since the I4790M mutation has also been implicated in diamide resistance in DBM, we screened for the presence of that mutation as well, but it was not detected in any of our samples (Table S6). Likewise, the potential site of the Y4891F mutation, identified in the Asiatic rice borer *Chilo suppressalis* [27,28], was captured by our reactions targeting the G4946E site, and there was no indication that this mutation was present in any of our samples (Table S7).

The results of the Kruskal–Wallis ANOVA for both mutation sites indicated significant differences between I4790K (H = 26.785, 9, *p* = 0.002) and G4946E (H = 27.514, 9, *p* = 0.001) frequencies within the sampled populations (Table S3). For the G4946E site, Dunn’s post hoc test indicated that only the MAN and NP colonies displayed significantly different frequencies of the mutation from the FRT susceptible colony, while the CSP colony was marginally different. For the I4790K site, only the GRD and BRK colonies were significantly different from the FRT susceptible colony. Spearman correlation analysis for each insecticide alongside the K/E allele frequency generated correlation coefficients detailing their relationships among all populations. The G4946E mutation frequency did not have a correlation with percent mortality induced by chlorantraniliprole (R = 0.177, *p* = 0.67) or cyantraniliprole (R = 0.116, *p* = 0.73). In comparison, the I4790K mutation frequency had a significant, negative correlation with percent mortality induced by chlorantraniliprole (R = −0.65, *p* = 0.03). While the I4790K mutation frequency had a negative correlation with cyantraniliprole-induced mortality, this was only marginally significant (R = −0.59, *p* = 0.06).

## 4. Discussion

Following the registration of the anthranilic diamide insecticide chlorantraniliprole for use on vegetable crops in the U.S. in the mid-2000s, it quickly became a major component of insecticide rotations for control of DBM on brassicaceous crops. Unfortunately, certain properties of this insecticide, particularly its remarkable efficacy and its persistence in the field, led to over-reliance on this chemistry, despite recommendations of insecticide rotations intended to prevent resistance development. As a result, local control failures with chlorantraniliprole were noted by 2016, and resistance was widespread throughout Florida and Georgia by 2018 [6,7,8]. Other diamides, such as cyantraniliprole and cyclaniliprole, had been registered in the mid-2010s but were not as extensively applied and so remained effective. Following widespread control failures with chlorantraniliprole, cyantraniliprole and cyclaniliprole became more prevalent in insecticide rotations for control of DBM on collards and other *Brassica* crops. As we documented previously and in more detail in this study, significant cross-resistance to chlorantraniliprole, cyantraniliprole, and cyclaniliprole was established in populations of DBM from Florida and Georgia by 2021 [8,36]. In the current study, we also documented the replacement of a target site mutation, G4946E, in the *PxRyR* that is the target of diamide insecticides, with a different mutation, I4790K, known to confer class-wide resistance to diamides.

Comparisons of the dose–response data for SMN and TIF (sampled in 2022) to our previously reported dose–response studies from the same region [6,35] revealed a temporal trend for diamide resistance development. The SMN population was highly resistant to chlorantraniliprole relative to the susceptible FRT laboratory colony (RR = 2942.7); however, relative to the T-S colony sampled in 2013, the RR was only 211, and resistance was almost unchanged relative to the field-derived populations assayed from 2018 [6,35] (Table 1). The same pattern was seen with the TIF population, which was highly resistant to chlorantraniliprole (RR = 511) relative to the FRT control, but only 36.7-fold resistant relative to the 2013 T-S population, and almost unchanged relative to the field-derived populations assayed from 2018 [6,35]. These data indicate that DBM populations from the Southeastern U.S. were already developing resistance to chlorantraniliprole by 2013, demonstrating a significant increase since then, with almost no increase from 2018 to 2022.

A much different pattern was seen with resistance to cyantraniliprole in the sampled DBM populations. The SMN population was highly resistant relative to the FRT control (RR = 1582), and equally resistant when compared to the 2013 T-S population (RR = 1417), indicating that the 2013 population was as susceptible as the FRT control (Table 1). Interestingly, cyantraniliprole resistance in the SMN population had increased 15 to 32-fold relative to the field-derived populations assayed in 2018. Again, the same pattern was found with the TIF population, which was 382-fold resistant compared to the FRT control, essentially identical to the 342-fold resistance relative to the T-S population (Table 1). Conversely, resistance to cyantraniliprole was 3.6 to 7.7-fold higher when compared to the field-derived populations collected during 2018. These data indicate that DBM populations from the Southeastern U.S. were fully susceptible to cyantraniliprole in 2013 but had developed low levels of resistance by 2018. By 2022, DBM populations from Georgia had become highly cross-resistant to both chlorantraniliprole and cyantraniliprole.

This temporal pattern of resistance development was recapitulated with the maximum dose assays. These assays expose population samples only to the highest permitted insecticide dose and so are less precise than dose–response experiments. However, they can be used to classify populations as susceptible (mortality ≥ 80%), intermediate (mortality 40–80%), or resistant (mortality ≤ 40%), and can be used to rapidly compare populations based on mortality counts [7,8]. Based on the results of the Kruskal–Wallis ANOVA comparing field populations to FRT, two populations assayed in 2018 (MAN and NP) were classified as resistant to chlorantraniliprole, and one (CSP) was classified as susceptible (Table 2). In contrast, all three of these 2018 populations were classified as susceptible to cyantraniliprole. All populations assayed from 2021 to 2023 were classified as resistant to chlorantraniliprole in comparison to FRT, except for the OKB population, which was non-significant by comparison (Table 2). However, the 2021–2023 populations were classified as resistant to cyantraniliprole, some highly so, as average mortality was less than 10% (Table 2). The results of the Kruskal–Wallis ANOVA indicated potential resistance to chlorantraniliprole at the maximum dose in comparison to the susceptible FRT colony in all colonies except for CSP and OKB (Table 2). Furthermore, this analysis indicated higher levels of resistance to cyantraniliprole at the maximum dose in colonies collected after 2021 (Table 2). Comparisons of colonies grouped by collection date verified the change from chlorantraniliprole resistance to diamide cross-resistance, as the colonies collected in 2018 were not significantly different from those collected in 2021–2023 regarding chlorantraniliprole mortality, but were significantly different regarding cyantraniliprole mortality (Table 3). Alongside the previously published dose–response data for the CSP, MAN, and NP colonies indicating high-level resistance to chlorantraniliprole with susceptibility to cyantraniliprole [35] (Table 1), these data indicate that resistance to chlorantraniliprole was well established before 2018, but cross-resistance to both chlorantraniliprole and cyantraniliprole only appeared later, between 2018 and 2021.

The temporal change in resistance to diamide insecticides raises the question of the underlying genetic changes in DBM populations from the Southeastern U.S. Specific mutations in the *PxRyR* that is the target of diamides, especially G4946E and I4790K or its variant I4790M, have been associated with resistance in DBM [14,15,16,17,18,19,21,22,23,24,35] and other insect pests [24,25,26,27,28,29,30,31,32,33,34], which prompted us to examine our populations for these mutations. In an earlier study [35], we used Sanger sequencing to show the presence of the G4946E mutation in the populations we sampled in 2018, but this method was not sufficient to precisely determine allele frequencies or to detect rare alleles. Therefore, the current study utilized an Illumina NextSeq approach to generate quantifiable data, as well as to identify potentially rare alleles in these populations. All three of our 2018 populations had a high proportion of G4946E reads, ranging from 75.0% (CSP) to 98.3% (MAN) (Figure 2, Tables S3 and S4). The CSP population retained a relatively high proportion of the susceptible wild type alleles (24.6% of total reads), which contrasts with the MAN and NP populations (1.4 and 5.7% wild type reads) and is consistent with the lower level of resistance to chlorantraniliprole in this population when compared to MAN and NP [35] (Figure 2, Tables S3 and S4). For 2018 populations, the I4790K mutation was detected only in the CSP population but accounted for just 2.7% of total reads (Figure 2, Tables S3 and S5). The two sampled populations (OMG and WRT) from 2021 had a marked reduction in the prevalence of the G4946E mutation (8.4 and 0.7% of the reads, respectively) and a noticeable increase in the prevalence of the I4790K mutation (9.3 and 18.0% of the reads, respectively) (Figure 2, Tables S3 and S5). Despite the relatively low estimates of the I4790K mutation, these two populations were also resistant to both chlorantraniliprole and cyantraniliprole at the maximum dose (Table 2). Continuing this trend, two populations (BRK and GRD) sampled in 2022 had significantly elevated proportions of the I4790K mutation compared to the FRT population, ranging from 76.0 to 84.0%, respectively. These populations also displayed very low proportions of G4946E, ranging from 0.2 to 3.9%, respectively (Figure 2, Tables S3 and S4). Once again, these populations were classified as resistant to both chlorantraniliprole and cyantraniliprole at the maximum dose (Table 2).

The changing prevalence of the *PxRyR* G4946E and I4790K mutations over time in the Southeastern U.S. can be associated with the functional aspects of these mutations, as well as the apparent changes in insecticide use patterns. Numerous studies have implicated the G4946E mutation with resistance to chlorantraniliprole in DBM populations. Jouraku et al. [15] compared two DBM strains, KU13 (with the G4946E mutation) and KA17 (with the I4790K mutation), for susceptibility to chlorantraniliprole, cyantranilipole, and the phthalic diamide, flubendiamide. The KU13 strain was highly resistant to chlorantraniliprole (RR > 20,000) but much less resistant to cyantraniliprole (RR = 678). In contrast, the KA17 strain was highly resistant to both chlorantraniliprole (RR > 20,000) and cyantraniliprole (RR = 66,200). Both strains were highly resistant to flubendiamide, such that an LC_50_ could not be determined. These results indicated that the G4946E mutation conferred resistance to chlorantraniliprole but was less effective against cyantraniliprole, and the I4790K mutation conferred resistance to both insecticides. This conclusion was reinforced by Jiang et al. [16], who introgressed the *PxRyR* 4946E, 4790K, and 4790M mutations into a common genetic background and assayed the effect on susceptibility to chlorantraniliprole, cyantriliprole, and three additional diamides (flubendiamide, tetraniliprole, and cyclaniliprole). The 4946E mutation conferred 54-fold resistance to chlorantraniliprole and 52-fold resistance to cyantraniliprole, as well as 39 to 739-fold resistance to the other diamides. In marked contrast, 4790K conferred 1620-fold resistance to chlorantraniliprole and 1762-fold resistance to cyantraniliprole, as well as >2000-fold resistance to the other diamides. On the other hand, 4790M was associated with low resistance to chlorantraniliprole and cyantraniliprole (21-fold and 16-fold, respectively), and similarly low resistance ratios for the other diamides. In a follow-up study, Jiang and coworkers [39] documented resistance ratios of 1857, 1433, and 2272-fold for chlorantraniliprole, cyantraniliprole, and flubendiamide, respectively, when comparing a susceptible strain to a CRISPR/Cas9 I4790K knock-in strain. Altogether, these studies establish that the I4790K mutation is significantly more effective than G4946E, as well as the mutant variant I4790M, in conferring resistance to cyantraniliprole, and to diamide insecticides as a class. Notably, the I4790M was not documented in any of our samples from this study, nor from our previous study [35]. It seems likely that this reduced effect of the I4790M mutation compared to I4790K accounts for the absence of I4790M in our sampled populations (Table S6).

Our study has demonstrated that resistance to chlorantraniliprole appeared not long after this diamide began to be extensively used for DBM control in the Southeastern U.S., and at least by 2018, this resistance was associated with the widespread presence of the G4946E mutation in the area. After widespread control failures with chlorantraniliprole, the use of other diamides, including cyantraniliprole and cyclaniliprole, increased, resulting in selection for resistance to these chemistries [8,36]. This study documents the appearance of high-level resistance to cyantraniliprole between 2018 and 2021, coincident with the replacement of the G4946E mutation with I4790K, suggesting that I4790K may be a major contributing mechanism to the development of diamide cross-resistance in the Southeastern U.S. As I4790K confers resistance to all currently registered active diamide ingredients, the resistance to both chlorantraniliprole and cyantraniliprole (and possibly cyclaniliprole, see [8]) represents cross-resistance and not independent resistance mechanisms.

It is noteworthy that the two populations sampled during 2021 (OMG and WRT) demonstrated resistance to both chlorantraniliprole and cyantraniliprole, but the prevalence of both the G4946E and I4790K mutations was not significantly different from the FRT population. This potentially indicates the presence of additional resistance mechanisms during the transition from G4946E prevalence to I4790K prevalence. Similarly, the prevalence of the two mutations in the LNG and OKB populations is almost identical, but LNG was more resistant to both chlorantraniliprole and cyantraniliprole in comparison to FRT, implying additional resistance mechanisms in the LNG population. The OKB population was collected from South–Central Florida, where *Brassica* crop production (and so DBM insecticide exposure) is seasonal. In contrast to South Georgia, where *Brassica* crop production is year-round, the selective pressure produced by insecticide applications is continuous. While the LNG site was a University of Georgia research plot where insecticide pressure was not continuous, it is surrounded by local commercial fields with constant insecticide pressure. Therefore, immigration of adult DBM from surrounding fields may have affected the resistance levels, as well as the associated resistance mechanisms present in the LNG population. Ultimately, the differences in exposure to diamides may select different types of resistance mechanisms in DBM populations. Although it is likely that target-site mutations of the RyR are major contributors to the observed diamide resistance in lepidopteran species, detoxification enzymes are a separate mechanism that have been consistently associated with insecticide resistance. Multiple genes across several gene families have been associated with diamide insecticide resistance in DBM populations, including the cytochrome P450s (*Cyp301a* and *Cyp9e2*) [40], uridine diphosphate-glucuronosyltransferases (*UGT33AA4*) [41], flavin-dependent monooxgenase (*FMO2*) [42], and glutathione S-transferases (GST) [43]. While these mechanisms have been well studied for DBM in response to chlorantraniliprole, little research has been conducted on the response to cyantraniliprole exposure in cross-resistant DBM populations. Such work could shed light on the potential contributions of detoxification enzymes in these diamide cross-resistant DBM populations and should be a focus of future studies on diamide resistance in DBM.

An additional question raised by our study is the reason for the marked reduction in prevalence of the G4946E mutation as I4790K emerged, resulting in the replacement, rather than the addition of I4790K alongside G4649E. We suggest a hypothesis that combining both mutations in the *PxRyR* compromises the function of the receptor and imposes an excessive fitness cost. The mutations are separated by an estimated 13–15 Å in a binding pocket in the RyR that is crucial for the diamide mechanism of action [21,23]. Molecular modeling indicates that the entrance of the pocket becomes narrower when the G4946E mutation is present, while the surface charge of the pocket becomes more negative, whereas with the I4790M mutation, the pocket becomes shallower since this site is located closer to the bottom of the pocket opening [23]. Clearly, both mutations affect the structure and overall charge of this region of the receptor, and these changes may adversely affect its function.

Interestingly, there is conflicting evidence regarding the fitness cost of the individual mutations. Fukada and coworkers [44] found no evidence that either mutation decreased longevity, number of eggs, hatching rate, or the number reaching the adult stage. However, the same study reported that the proportion of G4946E and I4790K declined in the field between *Brassica* growing seasons, or in the absence of diamide application, which implies a fitness cost under field conditions in the absence of insecticide selection. As an explanation, the authors of this study suggested that temperature may be one variable that could interact with target site mutation frequency in DBM populations. Furthermore, Pudasaini and coworkers [17] selected a susceptible DBM strain with chlorantraniliprole and tetraniliprole to produce two separate diamide-resistant strains under laboratory conditions. Subsequent to repeated selection, both strains had only the I4790K mutation and were cross-resistant to both chlorantraniliprole and tetraniliprole, as well as flubendiamide and cyantraniliprole. In addition, both strains were much more susceptible to spinosad, spinetoram, emamectin benzoate, diafenthiuron, chlorfenapyr, indoxacarb, and metaflumizone, indicating that the selection with the diamides did not increase resistance to these active ingredients. When diamide exposure was discontinued, both strains reverted to almost complete susceptibility within 10 generations. This rapid loss of resistance again implies a significant fitness cost associated with the I4790K mutation. This study also determined the haplotype and diplotype frequency of seven DBM field populations from Taiwan. The KG haplotype (with only the I4790K mutation) was most common, and KE haplotype (with both the I4790K and the G4946E) mutations were uncommon and detected in only three populations. The KG/KG diplotype was abundant in six of the seven populations, and the KG/KE diplotype was found in only three populations. Notably, the KE/KE diplotype, in which both alleles of the RyR gene contained both mutations, was rare (frequency ~ 5%) and found in only a single population. These results are consistent with the hypothesis that there is a significant fitness cost to combining both mutations in the same allele, especially in a homozygous condition. Although we did not determine the diplotype composition of our studied field populations, the proportions of the two mutations indicate that our 2018 samples were a combination of IE/IE and IE/IG, and our samples in 2022–2023 were mostly KG/KG or KG/IG.

This study is the first to document the presence of the *PxRyR* I4790K mutation in DBM from the Southeastern U.S., or indeed anywhere in North America. This also seems to be the first study to document a timeline of the replacement of the G4946E mutation with I4790K, and to associate that replacement with the development of widespread diamide cross-resistance in the area. The replacement of one target site mutation with a separate mutation at a different locus appears to be a novel finding. The rapid replacement, over a period of not more than four years, has significant implications for resistance management in DBM. For example, recent reports document high-level chlorantraniliprole resistance alongside moderate cyantraniliprole resistance in DBM populations from Arizona [45,46,47], a situation that is very similar to what the Southeastern U.S. experienced in the late 2010s [7]. Monitoring prevalence of the I4790K and G4946E mutations in such populations could inform management strategies to forestall the evolution of cross-resistance to additional diamides. As another example, in the Southeastern U.S., collards are started from greenhouse-grown transplants, which have been previously shown to spread insecticide-resistant DBM to new locations [48]. The use of diamide insecticides in transplant greenhouses could select for these mutations prior to shipment, which could result in diamide-resistant populations of DBM prior to the first field application. Monitoring populations for these mutations could guide insecticide rotations, such as prioritizing applications of diamides in the field as opposed to transplant greenhouses, to avoid inadvertent selection for cross-resistance.

## Figures and Tables

**Table 1:** Dose–response data for Georgia populations sampled from 2022 in comparison to results from Georgia populations sampled from 2018 and 2013.

Colony	Insecticide	LC_50_ mg ai/L ^a^	95% Fiducial Limits	N	Slope	Intercept	SE	x^2^	*p*	RR (FRT) ^b^	RR (CSP) ^c^	RR (NP) ^d^	RR (MAN) ^e^	RR (T-S) ^f^
SMN	Chlorantraniliprole	411.99	252.72–683.99	287	1.26	−3.31	0.22	32.1	<0.0001	2942.7	27.0	0.7	1.0	211.3
SMN	Cyantraniliprole	269.22	59.57–867.58	255	0.54	−1.31	0.12	17.4	<0.0001	1582.4	31.8	23.6	14.7	1416.9
TIF	Chlorantraniliprole	71.55	13.17–223.50	204	0.58	−1.07	0.15	13.9	0.0002	511.0	4.7	0.1	0.2	36.7
TIF	Cyantraniliprole	65.01	1.35–209.33	175	0.61	−1.10	0.22	7.2	0.0072	382.4	7.7	5.7	3.6	342.2

Resistance ratios were calculated by dividing the LC_50_ of our populations (SMN or TIF) by the LC_50_ being compared. ^a^ Data is represented as milligrams of active ingredient per liter (mg ai/L). ^b^ Resistance ratios for SMN and TIF colonies in comparison to the FRT colony. Dose–response data for FRT, CSP, NP, and MAN were previously published [35]. ^c^ Resistance ratios for SMN and TIF colonies in comparison to the CSP colony. ^d^ Resistance ratios for SMN and TIF colonies in comparison to the NP colony. ^e^ Resistance ratios for SMN and TIF colonies in comparison to the MAN colony. ^f^ Resistance ratios for SMN and TIF colonies in comparison to the 72 h response from the T-S collection from 2013 [6].

**Table 2:** Maximum dose–responses for ten diamondback moth populations to chlorantraniliprole and cyantraniliprole. Resistance level is classified as Susceptible (mortality ≥ 80%), Resistant (mortality < 40%), or Intermediate (mortality between 40 and 80%).

Population	Insecticide	Year	Percent Mortality	Z-Score	*p*	*p*-Adjusted	Resistance Level
FRT	Chlorantraniliprole	-	100.0	-	-	-	S
CSP	Chlorantraniliprole	2018	61.4	0.51	0.307	0.460	S
MAN	Chlorantraniliprole	2018	20.0	2.36	0.009	0.051 †	R
NP	Chlorantraniliprole	2018	26.0	2.00	0.022	0.078 †	R
OMG	Chlorantraniliprole	2021	20.7	2.43	0.007	0.056 †	R
WRT	Chlorantraniliprole	2021	13.7	2.79	0.002	0.059 †	R
LNG	Chlorantraniliprole	2022	20.0	2.41	0.008	0.051 †	R
GRD	Chlorantraniliprole	2022	17.5	2.56	0.005	0.057 †	R
BRK	Chlorantraniliprole	2022	11.7	3.03	0.001	0.053 †	R
OKB	Chlorantraniliprole	2023	34.5	1.30	0.095	0.253	S
FRT	Cyantraniliprole	-	100.0	-	-	-	S
CSP	Cyantraniliprole	2018	90.0	0.53	0.297	0.372	S
MAN	Cyantraniliprole	2018	87.5	0.68	0.248	0.338	S
NP	Cyantraniliprole	2018	72.0	1.08	0.139	0.241	S
OMG	Cyantraniliprole	2021	9.4	2.89	0.001	0.021 *	R
WRT	Cyantraniliprole	2021	3.3	3.22	0.001	0.028 *	R
LNG	Cyantraniliprole	2022	10.0	2.91	0.001	0.026 *	R
GRD	Cyantraniliprole	2022	10.0	2.83	0.002	0.020 *	R
BRK	Cyantraniliprole	2022	25.0	2.17	0.014	0.044 *	R
OKB	Cyantraniliprole	2023	33.3	1.82	0.034	0.085 †	R

* is indicative of significant differences (*p* < 0.05) in mortality according to Kruskal–Wallis One-Way ANOVA on Ranks followed by Dunn’s post hoc test compared to the susceptible FRT population, while † is indicative of marginal differences (*p* < 0.10). Each ANOVA was completed by insecticide, for chlorantraniliprole (H = 19.95, 9, *p* = 0.0182) and cyantraniliprole (H = 27.97, 9, *p* = 0.0009).

**Table 3:** Results of the Kruskal–Wallis One-Way ANOVA on ranks analyzing maximum dose bioassay responses of colonies based on date of collection.

Comparison	Z-Score	*p*	*p*-Adjusted
FRT Chlorantraniliprole vs. 2018 Chlorantraniliprole	2.09	0.035	0.053 †
FRT Cyantraniliprole vs. 2018 Cyantraniliprole	0.63	0.523	0.6042
FRT Chlorantraniliprole vs. 2021–2023 Chlorantraniliprole	3.18	0.001	0.003 *
FRT Cyantraniliprole vs. 2021–2023 Cyantraniliprole	3.57	<0.001	0.001 *
2018 Chlorantraniliprole vs. 2021–2023 Chlorantraniliprole	1.52	0.126	0.171
2018 Cyantraniliprole vs. 2021–2023 Cyantraniliprole	4.99	<0.001	<0.001 *

FRT represents the susceptible population. MAN, NP, and CSP are represented by “2018” and OMG, WRT, LNG, BRK, GRD, and OKB are represented by “2021–2023”. * is indicative of significant differences (*p* < 0.05) in mortality according to Kruskal–Wallis One-Way ANOVA on Ranks (H = 42.77, 5, <0.001) followed by Dunn’s post hoc test, while † is indicative of marginal differences (*p* < 0.10).

## Data Availability

Raw results of Illumina sequencing are given in Tables S1–S7. The raw data supporting the toxicological assays (dose/response and maximum dose assays of this article will be made available by the authors on request.

## Supplementary Materials

The following supporting information can be downloaded at: https://www.mdpi.com/article/10.3390/insects16111179/s1, Table S1: Primers used in the study. Table S2: PCR conditions used to generate the amplicons sequenced in this study. Table S3: Averaged allele frquencies for the G4946E and I4790K mutations, alongside the results of the Kruskal-Wallis test. Table S4: Additional information from the Illumina HiSeq Next Generation Sequencing. This table reports the total number of reads, as well as the total number of reads for each possible base, for each replicate at the 4946 site. These data represent the second base of the codon for both mutations and is only reflective of the estimated frequencies of the G4946E (GGG to GAG) mutation. Each replicate consisted of 20 pooled larvae or 40 possible alleles, so each allele could be expected to potentially contribute 2.5% of the reads. Reads less than 2% are assumed to be attributed to known Illumina background sequencing error. Table S5: Additional information from the Illumina HiSeq Next Generation Sequencing. This table reports the total number of reads, as well as the total number of reads for each possible base, for each replicate. These data represent the second base of the codon for both mutations and is only reflective of the estimated frequencies of the I4790K (ATA to AAA) mutation. Each replicate consisted of 20 pooled larvae or 40 possible alleles, so each allele could be expected to potentially contribute 2.5% of the reads. Reads less than 2% are assumed to be attributed to known Illumina background sequencing error. Table S6: Additional information from the Illumina HiSeq Next Generation Sequencing. This table reports the total number of reads, as well as the total number of reads for each possible base, for each replicate at the I4790 mutation site. These data represent the third base of the codon for the I4790 site and would only be reflective of the I4790M (ATA to ATG) variant mutation. Table S7: Additional information from the Illumina HiSeq Next Generation Sequencing. This table reports the total number of estimated reads, as well as the total number of estimated reads for each possible base, for each replicate at the Y4891 mutation site. These data represent the second base of the codon for the Y4891 site and would only be reflective of the Y4891F (TAC to TTC) variant mutation.
