# Supplementary material for: Temporal Variation in Target Site Mutations Is Associated with Diamide Cross-Resistance in Diamondback Moth Populations (Lepidoptera: Plutellidae) from Florida and Georgia, USA
Source: Insects. 2025 Nov 19;16(11):1179. doi: 10.3390/insects16111179 (PMC12653373; doi:10.3390/insects16111179)
Supplement: Supplementary file 1 [file insects-16-01179-s001.zip › insects-3926353-supplementary.pdf]

Supplementary Table S1. Primers used in the study.

Mutation	Primer Name	Primer Sequence
**G4946E**	GE-F	5′- GCGTCACGATCACAGACAAC -3′
**G4946E**	GE-RV	5′- CAGTGATGCTGCTCACCATC -3′
**I4790M/K**	IM-F	5′- ACGAAGACCCGATAGAGCTG -3′
**I4790M/K**	IM-RV	5′- GATGGTCTCTACATTGCGGAG -3′
**Illumina Overhang**	Forward	5′- TCGTCGGCAGCGTCAGATGTGTATAAGAGACAG -3′
**Illumina Overhang**	Reverse	5′- GTCTCGTGGGCTCGGAGATGTGTATAAGAGACAG -3′

Primers spanning each DBM RyR mutation site were designed using Primer3Plus.

Supplementary Table S2. PCR conditions used to generate the amplicons sequenced in this study.

Mutation	Method	Annealing Temperature (°C)	Extension times (s)	Product size (bp)	Number of Cycles
**G4946E**	Illumina	55	20	197	25
**I4790M/K**	Illumina	55	20	203	25

Supplementary Table S3. Averaged allele frquencies for the G4946E and I4790K mutations, alongside the results of the Kruskal-Wallis test.

Population	Mutation	Year	Mutation Percentage	Z-score	p	p-adjusted
**FRT**	G4946E	-	0.0	-	-	-
**CSP**	G4946E	2018	75.0	2.48	0.013	0.059†
**MAN**	G4946E	2018	98.3	3.24	0.001	0.027*
**NP**	G4946E	2018	94.0	3.00	0.002	0.030*
**OMG**	G4946E	2021	8.4	1.99	0.047	0.140
**WRT**	G4946E	2021	0.7	0.42	0.670	0.773
**LNG**	G4946E	2022	1.4	1.04	0.298	0.479
**GRD**	G4946E	2022	3.9	1.70	0.088	0.220
**BRK**	G4946E	2022	0.2	0.00	1.000	1.000
**OKB**	G4946E	2023	0.4	0.31	0.758	0.832
**FRT**	I4790K	-	0.0	-	-	-
**CSP**	I4790K	2018	2.7	0.615	0.539	0.693
**MAN**	I4790K	2018	0.1	0.00	1.000	1.000
**NP**	I4790K	2018	0.1	0.00	1.000	1.000
**OMG**	I4790K	2021	9.3	1.51	0.130	0.256
**WRT**	I4790K	2021	18.0	1.70	0.088	0.233
**LNG**	I4790K	2022	34.1	2.01	0.044	0.143
**GRD**	I4790K	2022	84.0	3.19	0.001	0.017*
**BRK**	I4790K	2022	76.0	3.05	0.002	0.017*
**OKB**	I4790K	2023	37.0	2.10	0.035	0.143

Results of the Kruskal-Wallis ANOVA for G4946E (H=27.50, 9, 0.001) and I4790K (H=26.80, 9, 0.001). * is indicative of significant differences ($p < 0.05$) in mortality according to Kruskal-Wallis One-Way ANOVA on Ranks followed by Dunn's post hoc test, while † is indicative of marginal differences ($p < 0.10$).

Supplementary Table S4. Additional information from the Illumina HiSeq Next Generation Sequencing. This table reports the total number of reads, as well as the total number of reads for each possible base, for each replicate at the 4946 site. These data represent the second base of the codon for both mutations and is only reflective of the estimated frequencies of the G4946E (GGG to GAG) mutation. Each replicate consisted of 20 pooled larvae or 40 possible alleles, so each allele could be expected to potentially contribute 2.5% of the reads. Reads less than 2% are assumed to be attributed to known Illumina background sequencing error.

Colony	Replicate	Amino Acid Position (Base X**X**X)	Total Number of Reads	Number of A Reads	Number of C Reads	Number of G Reads	Number of T Reads
FRT	1	4946	3,758,239	1,198 (0.03%)	2,128 (0.06%)	3,748,961 (99.75%)	5,952 (0.16%)
FRT	2	4946	4,045,341	1,356 (0.03%)	2,137 (0.05%)	4,035,930 (99.77%)	5,918 (0.15%)
FRT	3	4946	3,429,698	1,199 (0.03%)	1,781 (0.05%)	3,421,982 (99.78%)	4,736 (0.14%)
MAN	1	4946	3,449,847	3,329,452 (96.51%)	1,448 (0.04%)	114,070 (3.31%)	4,877 (0.14%)
MAN	2	4946	2,983,451	2,962,446 (99.30%)	1,495 (0.05%)	14,634 (0.49%)	4,876 (0.16%)
MAN	3	4946	2,948,159	2,922,618 (99.13%)	1,611 (0.06%)	19,560 (0.66%)	4,370 (0.15%)
NP	1	4946	3,472,673	3,046,890 (87.74%)	1,462 (0.04%)	419,091 (12.07%)	5,230 (0.15%)
NP	2	4946	3,167,555	3,012,010 (95.09%)	1,460 (0.05%)	149,897 (4.73%)	4,187 (0.13%)
NP	3	4946	3,037,341	3,020,013 (99.43%)	1,326 (0.04%)	11,592 (0.38%)	4,410 (0.15%)
CSP	1	4946	3,479,604	2,798,842 (80.44%)	2,057 (0.06%)	671,894 (19.31%)	6,811 (0.19%)
CSP	2	4946	3,727,838	2,725,074 (73.10%)	1,713 (0.05%)	995,607 (26.71%)	5,444 (0.14%)
CSP	3	4946	3,247194	2,329,246 (71.73%)	2,309 (0.07%)	908,045 (27.96%)	7,594 (0.24%)
OMG	1	4946	3,042,363	110,740 (3.64%)	1,583 (0.05%)	2,923,335 (96.09%)	6,705 (0.22%)
OMG	2	4946	2,922,514	372,748 (12.76%)	1,440 (0.05%)	2,542,136 (86.98%)	6,190 (0.21%)
OMG	3	4946	3,113,120	280,553 (9.01%)	1,811 (0.06%)	2,823,588 (90.70%)	7,167 (0.23%)
WRT	1	4946	3,024,487	1,625 (0.05%)	1,708 (0.06%)	3,013,616 (99.64%)	7,538(0.25%)
WRT	2	4946	2,894,518	1,812 (0.06%)	1,222 (0.04%)	2,885,735 (99.70%)	5,749 (0.20%)
WRT	3	4946	3,299,195	65,007 (1.97%)	1,684 (0.05%)	3,225,231 (97.76%)	7,273 (0.22%)
LNG	1	4946	3,203,217	45,418 (1.42%)	1,563 (0.05%)	3,150,842 (98.36%)	5,394 (0.17%)
LNG	2	4946	2,884,358	46,276 (1.60%)	1,053 (0.04%)	2,832,262 (98.19%)	4,767 (0.17%)
LNG	3	4946	2,741,890	35,730 (1.30%)	1,141 (0.04%)	2,699,618 (98.46%)	5,401 (0.20%)
BRK	1	4946	2,732,464	3,995 (0.15%)	1,375 (0.05%)	2,721,413 (99.60%)	5,681 (00.20%)
BRK	2	4946	2,500,065	8,707 (0.35%)	886 (0.03%)	2,486,802 (99.47%)	3,670 (0.15%)

BRK	3	4946	2,747,934	3,577 (0.13%)	1,430 (0.05%)	2,737,042 (99.61%)	5,885 (0.22%)
GRD	1	4946	2,739,748	95,792 (3.50%)	1,112 (0.04%)	2,638,620 (96.31%)	4,224 (0.15%)
GRD	2	4946	2,867,485	106,904 (3.73%)	1,392 (0.05%)	2,753,135 (96.01%)	6,054 (0.21%)
GRD	3	4946	3,138,601	143,402 (4.57%)	1,489 (0.05%)	2,987,388 (95.18%)	6,322 (0.20%)
OKB	1	4946	3,095,422	1,559 (0.05%)	1,665 (0.05%)	3,085,193 (99.67%)	7,005 (0.23%)
OKB	2	4946	2,783,041	34,186 (1.23%)	1,058 (0.04%)	2,742,999 (98.56%)	4,798 (0.17%)
OKB	3	4946	2,641,806	1,207 (0.05%)	1,265 (0.05%)	2,633,617 (99.69%)	5,717 (0.21%)

Supplementary Table S5. Additional information from the Illumina HiSeq Next Generation Sequencing. This table reports the total number of reads, as well as the total number of reads for each possible base, for each replicate. These data represent the second base of the codon for both mutations and is only reflective of the estimated frequencies of the I4790K (ATA to AAA) mutation. Each replicate consisted of 20 pooled larvae or 40 possible alleles, so each allele could be expected to potentially contribute 2.5% of the reads. Reads less than 2% are assumed to be attributed to known Illumina background sequencing error.

Colony	Replicate	Amino Acid Position (Base X**X**X)	Total Number of Reads	Number of A Reads	Number of C Reads	Number of G Reads	Number of T Reads
FRT	1	4790	2,950,853	3,031 (0.10%)	309 (0.01%)	10,465 (0.35%)	2,937,048 (99.53%)
FRT	2	4790	2,769,164	2,258 (0.08%)	229 (0.01%)	8,157 (0.29%)	2,758,520 (99.62%)
FRT	3	4790	2,680,864	2,546 (0.09%)	274 (0.01%)	9,364 (0.35%)	2,668,680 (99.55%)
MAN	1	4790	2,665,337	6,272 (0.23%)	456 (0.02%)	30,689 (1.15%)	2,627,920 (98.60%)
MAN	2	4790	2,632,815	2,455 (0.09%)	275 (0.01%)	27,216 (1.03%)	2,602,869 (98.87%)
MAN	3	4790	2,380,259	4,530 (0.19%)	325 (0.01%)	25,072 (1.05%)	2,350,332 (99.75%)
NP	1	4790	2,540,116	5,006 (0.20%)	382 (0.01%)	9,528 (0.38%)	2,525,200 (99.41%)
NP	2	4790	3,052,003	2,553 (0.08%)	407 (0.01%)	32,326 (1.06%)	3,016,717 (98.85%)
NP	3	4790	2,795,945	2,490 (0.09%)	238 (0.01%)	27,541 (0.98%)	2,765,676 (98.92%)
CSP	1	4790	3,171,759	140,395 (4.43%)	1,006 (0.03%)	30,409 (0.96%)	2,999,949 (94.58%)
CSP	2	4790	2,875,872	3,020 (0.10%)	157 (0.01%)	28,661 (1.0%)	2,844,034 (98.89%)
CSP	3	4790	2,969,210	110,500 (3.72%)	785 (0.03%)	29,301 (0.99%)	2,828,624 (95.26%)

OMG	1	4790	3,487,591	294,050 (8.43%)	2,059 (0.06%)	21,875 (0.63%)	3,169,607 (90.88%)
OMG	2	4790	2,703,555	254,339 (9.41%)	1,699 (0.06%)	16,539 (0.61%)	2,430,978 (89.92%)
OMG	3	4790	2,765,191	283,619 (10.26%)	2,131 (0.08%)	18,320 (0.66%)	2,461,121 (89%)
WRT	1	4790	3,312,592	1,228,111 (37.08%)	7,363 (0.22%)	14,329 (0.43%)	2,062,789 (62.27%)
WRT	2	4790	2,937,298	297,701 (10.14%)	2,074 (0.07%)	18,472 (0.63%)	2,619,051 (89.16%)
WRT	3	4790	3,749,000	253,368 (6.76%)	1,861 (0.05%)	22,275 (0.59%)	3,471,896 (92.60%)
LNG	1	4790	3,213,954	1,723,694 (53.63%)	10,297 (0.32%)	16,202 (0.50%)	1,463,761 (45.55%)
LNG	2	4790	2,645,418	129,322 (4.89%)	979 (0.04%)	5,159 (0.19%)	2,509,958 (94.88%)
LNG	3	4790	3,280,834	1,438,773 (43.86%)	8,569 (0.26%)	19,036 (0.58%)	1,814,456 (55.30%)
BRK	1	4790	2,503,074	2,039,986 (81.50%)	11,931 (0.48%)	5,019 (0.20%)	446,138 (17.82%)
BRK	2	4790	2,661,347	2,011,347 (75.57%)	11,903 (0.45%)	7,126 (0.26%)	631,427 (23.72%)
BRK	3	4790	2,966,325	2,104,310 (70.94%)	12,163 (0.41%)	7,427 (0.25%)	842,425 (28.40%)
GRD	1	4790	3,005,797	2,701,069 (89.86%)	15,602 (0.52%)	3,092 (0.10%)	286,034 (9.52%)
GRD	2	4790	3,593,401	2,400,729 (66.81%)	14,210 (0.40%)	12,703 (0.35%)	1,165,759 (32.44%)
GRD	3	4790	3,079,802	2,932,983 (95.23%)	18,070 (0.59%)	1,874 (0.06%)	126,875 (4.12%)
OKB	1	4790	3,012,620	1,031,552 (34.24%)	6,102 (0.20%)	15,067 (0.50%)	1,959,899 (65.06%)
OKB	2	4790	3,022,560	1,061,425 (35.12%)	6,355 (0.21%)	13,018 (0.43%)	1,941,762 (64.24%)
OKB	3	4790	3,604,655	1,512,565 (41.96%)	9,425 (0.26%)	14,895 (0.41%)	2,067,770 (57.37%)

Supplementary Table S6. Additional information from the Illumina HiSeq Next Generation Sequencing. This table reports the total number of reads, as well as the total number of reads for each possible base, for each replicate at the I4790 mutation site. These data represent the third base of the codon for the I4790 site and would only be reflective of the I4790M (ATA to ATG) variant mutation.

Colony	Replicate	Amino Acid Position (Base XX**X**)	Total Number of Reads	Number of A Reads	Number of C Reads	Number of G Reads	Number of T Reads
FRT	1	4790	2,950,846	2,950,265 (100%)	8 (0%)	203 (0%)	370 (0%)
FRT	2	4790	2,769,172	2,768,708 (100%)	7 (0%)	144 (0%)	313 (0%)
FRT	3	4790	2,680,838	2,680,354 (100%)	16 (0%)	165 (0%)	303 (0%)
MAN	1	4790	2,665,336	2,664,427 (100%)	42 (0%)	191 (0%)	676 (0%)
MAN	2	4790	2,632,851	2,631,966 (100%)	25 (0%)	209 (0%)	651 (0%)
MAN	3	4790	2,380,284	2,379,419 (100%)	124 (0%)	174 (0%)	567 (0%)
NP	1	4790	2,540,113	2,539,288 (100%)	58 (0%)	188 (0%)	579 (0%)
NP	2	4790	3,052,040	3,050,977 (100%)	55 (0%)	233 (0%)	775 (0%)
NP	3	4790	2,795,925	2,794,902 (100%)	32 (0%)	265 (0%)	726 (0%)
CSP	1	4790	3,171,823	3,170,856 (100%)	58 (0%)	177 (0%)	732 (0%)
CSP	2	4790	2,875,871	2,875,099 (100%)	37 (0%)	162 (0%)	573 (0%)
CSP	3	4790	2,969,230	2,968,404 (100%)	62 (0%)	169 (0%)	595 (0%)
OMG	1	4790	3,487,606	3,486,316 (100%)	73 (0%)	465 (0%)	752 (0%)
OMG	2	4790	2,703,560	2,702,702 (100%)	66 (0%)	317 (0%)	475 (0%)
OMG	3	4790	2,765,198	2,764,331 (100%)	77 (0%)	316 (0%)	474 (0%)
WRT	1	4790	3,313,088	3,311,771 (100%)	269 (0%)	583 (0%)	465 (0%)
WRT	2	4790	2,937,306	2,936,481 (100%)	82 (0%)	231 (0%)	512 (0%)
WRT	3	4790	3,749,406	3,747,525 (100%)	256 (0%)	949 (0%)	676 (0%)
LNG	1	4790	3,213,979	3,213,093 (100%)	272 (0%)	192 (0%)	422 (0%)
LNG	2	4790	2,645,419	2,644,651 (100%)	40 (0%)	287 (0%)	441 (0%)
LNG	3	4790	3,280,866	3,279,901 (100%)	273 (0%)	149 (0%)	543 (0%)
BRK	1	4790	2,503,104	2,502,307 (100%)	387 (0%)	155 (0%)	255 (0%)
BRK	2	4790	2,661,827	2,661,051 (100%)	361 (0%)	123 (0%)	292 (0%)
BRK	3	4790	2,966,349	2,965,520 (100%)	374 (0%)	154 (0%)	301 (0%)
GRD	1	4790	3,005,833	3,004,852 (100%)	436 (0%)	240 (0%)	305 (0%)

GRD	2	4790	3,593,441	3,592,266 (100%)	415 (0%)	236 (0%)	524 (0%)
GRD	3	4790	3,079,882	3,078,953 (100%)	484 (0%)	204 (0%)	241 (0%)
OKB	1	4790	3,012,777	3,011,833 (100%)	267 (0%)	260 (0%)	417 (0%)
OKB	2	4790	3,022,733	3,021,470 (100%)	192 (0%)	421 (0%)	650 (0%)
OKB	3	4790	3,604,741	3,603,572 (100%)	323 (0%)	308 (0%)	538 (0%)

Supplementary Table S7. Additional information from the Illumina HiSeq Next Generation Sequencing. This table reports the total number of estimated reads, as well as the total number of estimated reads for each possible base, for each replicate at the Y4891 mutation site. These data represent the second base of the codon for the Y4891 site and would only be reflective of the Y4891F (TAC to TTC) variant mutation.

Colony	Replicate	Amino Acid Position (Base X**X**X)	Total Estimated Number of Reads	Estimated Number of A Reads	Estimated Number of C Reads	Estimated Number of G Reads	Estimated Number of T Reads
FRT	1	4922	3,298,479	3,297,275 (100%)	139 (0%)	209 (0%)	856 (0%)
FRT	2	4922	3,549,631	3,548,377 (100%)	159 (0%)	211 (0%)	884 (0%)
FRT	3	4922	3,006,072	3,004,994 (100%)	118 (0%)	216 (0%)	744 (0%)
MAN	1	4922	3,029,342	3,028,010 (100%)	58 (0%)	215 (0%)	1,059 (0%)
MAN	2	4922	2,619,971	2,618,665 (100%)	100 (0%)	233 (0%)	973 (0%)
MAN	3	4922	2,588,551	2,587,386 (100%)	77 (0%)	27 (0%)	831 (0%)
NP	1	4922	3,048,654	3,047,257 (100%)	86 (0%)	189 (0%)	1,122 (0%)
NP	2	4922	2,782,239	2,781,146 (100%)	57 (0%)	182 (0%)	854 (0%)
NP	3	4922	2,669,261	2,668,060 (100%)	71 (0%)	239 (0%)	891 (0%)
CSP	1	4922	3,055,170	3,053,728 (100%)	166 (0%)	212 (0%)	1,064 (0%)
CSP	2	4922	3,269,901	3,268,654 (100%)	90 (0%)	219 (0%)	938 (0%)
CSP	3	4922	2,851,231	2,849,864 (100%)	81 (0%)	215 (0%)	1,071 (0%)
OMG	1	4922	2,672,723	2,671,614 (100%)	77 (0%)	178 (0%)	853 (0%)
OMG	2	4922	2,568,409	2,567,471 (100%)	47 (0%)	131 (0%)	759 (0%)
OMG	3	4922	2,734,685	2,733,650 (100%)	83 (0%)	188 (0%)	764 (0%)
WRT	1	4922	2,657,462	2,656,412 (100%)	72 (0%)	189 (0%)	789 (0%)
WRT	2	4922	2,543,522	2,542,521 (100%)	79 (0%)	135 (0%)	787 (0%)
WRT	3	4922	2,897,182	2,896,234 (100%)	55 (0%)	158 (0%)	735 (0%)
LNG	1	4922	2,812,909	2,812,024 (100%)	102 (0%)	163 (0%)	620 (0%)
LNG	2	4922	2,534,141	2,533,124 (100%)	82 (0%)	164 (0%)	771 (0%)
LNG	3	4922	2,409,175	2,408,358 (100%)	77 (0%)	139 (0%)	601 (0%)
BRK	1	4922	2,400,197	2,399,175 (100%)	71 (0%)	185 (0%)	766 (0%)
BRK	2	4922	2,198,588	2,197,781 (100%)	54 (0%)	140 (0%)	613 (0%)
BRK	3	4922	2,413,217	2,412,214 (100%)	99 (0%)	180 (0%)	724 (0%)
GRD	1	4922	2,405,375	2,404,392 (100%)	62 (0%)	178 (0%)	743 (0%)

GRD	2	4922	2,517,120	2,516,195 (100%)	79 (0%)	195 (0%)	651 (0%)
GRD	3	4922	2,756,405	2,755,273 (100%)	85 (0%)	202 (0%)	845 (0%)
OKB	1	4922	2,718,226	2,717,059 (100%)	84 (0%)	176 (0%)	907 (0%)
OKB	2	4922	2,444,726	2,443,756 (100%)	70 (0%)	166 (0%)	734 (0%)
OKB	3	4922	2,322,262	2,321,423 (100%)	64 (0%)	163 (0%)	612 (0%)
